# Comparative study of bovine and synthetic hydroxyapatite in micro- and nanosized on osteoblasts action and bone growth

**DOI:** 10.1371/journal.pone.0311652

**Published:** 2025-01-24

**Authors:** Maria Apriliani Gani, Gyubok Lee, Chrismawan Ardianto, Fedik Abdul Rantam, Maria Lucia Ardhani Dwi Lestari, Rafi Addimaysqi, I Ketut Adnyana, Kangwon Lee, Junaidi Khotib

**Affiliations:** 1 Doctoral Programme of Pharmaceutical Sciences, Faculty of Pharmacy, Airlangga University, Surabaya, Indonesia; 2 Department of Pharmacology-Clinical Pharmacy, School of Pharmacy, Bandung Institute of Technology, Bandung, Indonesia; 3 Bioscience and Biotechnology Research Center, Bandung Institute of Technology, Bandung, Indonesia; 4 Department of Applied Bioengineering, Research Institute for Convergence Science, Seoul National University, Seoul, Republic of Korea; 5 Department of Pharmacy Practice, Faculty of Pharmacy, Airlangga University, Surabaya, Indonesia; 6 Department of Microbiology, Faculty of Veterinary Medicine, Airlangga University, Surabaya, Indonesia; 7 Department of Pharmaceutical Science, Faculty of Pharmacy, Airlangga University, Surabaya, Indonesia; 8 Faculty of Medicine, Airlangga University, Surabaya, Indonesia; 9 Research Institute for Convergence Science, Seoul National University, Suwon, Republic of Korea; Università degli Studi della Campania, ITALY

## Abstract

Hydroxyapatite (HA) is widely used as a bone graft. However, information on the head-to-head osteoinductivity and in vivo performance of micro- and nanosized natural and synthetic HA is still lacking. Here, we fabricated nanosized bovine HA (nanoBHA) by using a wet ball milling method and compared its in vitro and in vivo performance with microsized BHA, nanosized synthetic HA (nanoHA), and microsized synthetic HA (HA). The results showed that the wet ball milling method successfully reduced the particle size of BHA to 40 nm without changing its natural characteristics. NanoBHA was able to maintain cell viability and induce cell proliferation and calcium deposits. NanoBHA promoted osteogenic differentiation via OPN as a specific regulator, with a 13-fold greater expression level. NanoBHA and HA also activated ERK1/2 indicated corresponding to the proliferation-differentiation and death of cells, respectively. The calvarial bone defect model showed that nanoBHA induced bone growth based on CT images, which is in line with the histological results showing the presence of bone cells and connective tissue at the nanoBHA implantation site. In conclusion, natural HA outperformed synthetic HA. Our findings will attract interest in further research into nanomaterials and their mechanism of action in bone remodeling.

## 1. Introduction

The bones provide overall mechanical stability and mobility and protect vital organs [1]. In skeletal trauma and bone diseases that result in defects, the normal function of the bone is altered, which negatively impacts patients’ quality of life [2]. Over 2.2 million patients worldwide require surgical procedures to treat bone defects annually [3]. Thus, with the rapid development of tissue engineering, researchers are developing therapeutic strategies to accelerate bone defect healing. Among these strategies, bone grafting is used to attach certain materials to the defect site. This is useful for replacing and speeding up the healing process of defective bone tissues [4]. Autologous bone is a popular material for bone grafting. It has been shown to induce bone growth and is considered as the gold standard in clinical practice [4]. However, autologous bone has limited availability, and its use may result in tissue morbidity at the bone donor site. Another drawback is the complexity of bone grafting, which requires two operations to take and implant the necessary bone [4, 5].

The mineral part of the bone that interacts with body fluids actively contributes to homeostasis processes. This feature is imparted by the two main components of the bony matrix: nonstoichiometric hydroxyapatite (HA), which accounts for 70% of bone weight, and collagen, which accounts for 20% [1, 6]. HA has been widely used as a biomaterial in bone tissue regeneration because of its close resemblance to human bones [7]. As a biomaterial, HA has been shown to have biocompatible, biodegradable, osteoconductive, and osteoinductive properties [7]. HA is obtained through synthesis from chemical precursors such as calcium nitrate tetrahydrate and diammonium hydrogen phosphate [8] or extraction from natural resources such as bovine bone [9]. Synthetic and natural HA have similar diffraction patterns [10]. However, synthetic HA does not have trace elements that resemble the nonstoichiometric HA present in human bones [11]. In our previous study, we found that bovine HA (BHA) bone graft showed osteoconductive properties compared with synthetic HA bone graft [12]. Moreover, the BHA bone graft induced bone growth and accelerated the inflammatory phase of bone healing by increasing the expression of CD163 [12].

Nanotechnology and nanomaterials have developed tremendously over the last two decades. Nanomaterials have at least three advantages in the field of bone tissue engineering. First, nanomaterials have a large surface area, which increases the material’s wettability, facilitating its interaction with bone tissue [13]. Second, nanomaterials, including nanosized HA, mimic the size of nonstoichiometric HA present in bone tissue, which has a grain size of 50–200 nm [14, 15]. Third, nanomaterials may have specific interactions with membrane proteins, which will provide new insights into how biomaterials interact with bone cells [16, 17]. In another study, we reduced the particle size of BHA (from ~5 μm to ~1 μm) using a dry ball milling method for 9 h. However, the method was ineffective for producing nanomaterials because no media were used in the milling process [18]. Other studies used process control agents (PCAs) as milling media to reduce the particle size of natural HA to the nanoscale. However, PCAs contaminate the obtained nanomaterials by changing their morphology and elemental distribution [19, 20]. Here, we used deionized water as nonhazardous milling media to reduce the particle size of BHA to fabricate nanosized BHA (nanoBHA). A decrease in the particle size of BHA indicates an increase in the material’s osteoinductive property [13–15]. Although the osteoconductive property of BHA was reported in our previous study [12], its osteoinductive property has not yet been investigated. Here, we compared nanoBHA with BHA, nanosized synthetic HA (nanoHA), and HA to examine the effects of micro- and nanosized natural and synthetic HA in bone regeneration.

In this context, the present study aimed to i) fabricate nanoBHA by using the nonhazardous wet ball milling method; ii) characterize and compare nanoBHA with BHA, nanoHA, and microsized synthetic HA (HA) from a physicochemical standpoint using various techniques; iii) evaluate their effects on preosteoblasts in terms of viability, proliferation, differentiation, calcium deposition, and the involvement of ERK1/2 signaling in cells; and iv) evaluate their performance in calvarial bone defects in Wistar rats.

## 2. Materials and methods

### 2.1 Fabrication of NanoBHA

BHA was extracted in accordance with our previously developed method [9]. A novel wet ball milling method was used to reduce the particle size of BHA to the nanoscale. Briefly, a pre-heated 0.4% gelatin solution was mixed with BHA powder, stirred, and sonicated for 3 h. Moreover, yttrium milling balls (size, 0.3 mm; Chemco, China) were added to the mixture, stirred at 1000 rpm for 6 h in an ice bath, and filtered. Then, the nanoBHA suspension was used for the in vitro study. For the in vivo study, the nanoBHA suspension was formulated as a powder by mixing nanoBHA and trehalose in a 9:1 ratio and lyophilized for 48 h.

### 2.2 Characterization of materials

NanoBHA, nanoHA, BHA, and HA were characterized to examine their physical and chemical characteristics. The particle size and zeta potential value were examined by using dynamic light scattering (DLS; Zetasizer Nano Series, Malvern PANalytical Ltd., UK). The surface morphology and elemental percentage were examined using scanning electron microscopy–energy dispersive X-ray analysis (EVO MA 10, Carl Zeiss, Germany). The materials’ grain size was examined using transmission electron microscopy (JEM-3010, JEOL, Japan). X-ray diffraction (XRD) and Fourier transform infrared spectroscopy (FTIR) peaks were observed using an X-ray diffractometer (X’Pert Pro, Malvern PANalytical Ltd.) and FTIR spectrometer (Alpha, Bruker, USA), respectively.

### 2.3 Cell culture

MC3T3-E1 cell line subclone 4 (CRL-2593, ATCC, USA) was used as the preosteoblast model in vitro. The cells were cultured in growth medium (α-MEM [LM008-53, Welgene, South Korea] supplemented with 10% fetal bovine serum [Cellsera, Australia] and 1% penicillin-streptomycin [LS 202–02, Welgene]). The medium used to induce differentiation was a growth medium supplemented with 50 μg mL^−1^ ascorbic acid (255564, Sigma-Aldrich, USA), 10 mM β-glycerophosphate (G9422, Sigma-Aldrich), and 100 nM dexamethasone (D4902, Sigma-Aldrich). The medium was changed every three days. The cells were grown at 37°C under 5% CO_2_ and subcultured with 0.125% trypsin–ethylenediaminetetraacetic acid (25200–056, Gibco, USA) when they reached 80% confluency.

### 2.4 Lactate Dehydrogenase (LDH) release assay

Sections 2.4 and 2.5 aimed to ensure that NanoBHA, NanoHA, BHA, and HA are non-toxic to preosteoblast cells. The cells were seeded in 96-well plates at a density of 4000 cells for 24 h. Then, they were treated with each material with a final concentration of 50 μg mL^−1^ and incubated for 24 h. Subsequently, the cells were reacted with LDH kit (BCT-LDHP1000, Biomax, South Korea) in accordance with the manufacturer’s instructions. Finally, the plate was read using a microplate reader (Synergy H1; Hybrid reader, USA) at a wavelength of 490 nm.

### 2.5 Viability assay

The cells were seeded in the same condition as Section 2.4. Thereafter, each well was added with 10 μL of Cell Counting Kit-8 (CCK-8) reagent (#EZ-3000, DoGenBio, South Korea) and incubated for 2 h. Subsequently, the plates were read using a microplate reader at a wavelength of 450 nm.

### 2.6 Live/dead assay

Live/dead assay aimed to ensure that the differentiation conditions did not interfere with cells’ viability. The cells were seeded at 2 × 10^4^ in 24-well plates and grown until confluence. Afterward, cell differentiation was induced. The growth medium was changed to differentiation medium and cells were treated with each material with a final concentration of 50 μg mL^−1^ which was mixed with medium. The negative control group was cells that were grown in growth medium and received no material treatment, while the growth factor group was cells grown in differentiation medium only. After 4, 7, and 14 days of differentiation induction, the cells were washed and reacted using the LIVE/DEAD^™^ Kit (L3224, Invitrogen, USA). Each well was added with a mixture containing 4 mM calcein AM and 2 mM ethidium homodimer-1 and incubated for 30 min at room temperature (RT) without light. Subsequently, the cells were observed under a fluorescence microscope (Carl Zeiss, Germany).

### 2.7 Proliferation assay

In contrast to the viability experiments, the proliferation assay used a lower seeding density of 500 cells per 96-well plate to enable longer-term cell growth. Proliferation was not only checked for MC3T3-E1 cells (preosteoblasts) but also for the cells that grew for 14 days in the differentiation inducement condition (induced osteoblasts). After 24 h of 24 h, cells were treated with each material with a final concentration of 50 μg mL^−1^ until 1, 4, and 7 days. Proliferation was then checked by adding 10 μL of CCK-8 reagent (#EZ-3000, DoGenBio) followed by 2 h of incubation, and the plates were read using a microplate reader at a wavelength of 450 nm.

### 2.8 Alkaline Phosphatase (ALP) staining

The cells were induced to differentiate in the same condition as Section 2.6. After 1, 4, 7, and 14 days, the cells were fixed with 4% paraformaldehyde for 15 min. Each well was added with 5-bromo-4-chloro-3-indolyl phosphate/nitro blue tetrazolium (#B1911, Sigma-Aldrich), incubated for 1 h without light, and then washed. Macroscopic images were taken using a digital camera, whereas microscopic images were taken using a light microscope. The percentage of the stained area of wells was quantified using ImageJ version 1.53k (NIH, USA).

### 2.9 Alizarin red staining

Similar to Section 2.6, cell differentiation was induced. After 14 days, the cells were fixed with 4% paraformaldehyde, treated with Alizarin Red solution (pH 4,4) for 30 min at RT, and washed. Thereafter, the red color indicating positive test results was extracted. The cells were treated with acetic acid for 30 min, heated to 85°C for 10 min, and centrifugated at 20000 *g* for 15 min. Next, the supernatant was obtained and mixed with 10% ammonium hydroxide. Finally, the plate was read using a microplate reader at a wavelength of 405 nm.

### 2.10 F-actin staining

Similar to Section 2.6, cell differentiation was induced. After 1 h, 1 day, and 14 days, the cells were subcultured to observe a more definite morphology of each single cell. After that cells were fixed with 4% paraformaldehyde, permeabilized with 0.1% Triton X-100 for 5 min, washed, blocked with 1% bovine serum albumin (BSA) at RT for 15 min, and washed again. Then, the cells were added with Alexa Fluor^®^ 488 Phalloidin (#8878S, Molecular Probes, USA) for 1 h at RT and washed. Subsequently, the cells were added with 4′,6-diamidino-2-phenylindole for 5 min and washed. Cell layers were observed under a fluorescence microscope.

### 2.11 Quantitative real-time reverse transcription Polymerase Chain Reaction (PCR)

Similar to Section 2.6, cell differentiation was induced. After 4, 7, and 14 days, total RNA was isolated using the TRIzol method (#15596026, Thermo Scientific), and cDNA was synthesized by using the SuperScript^™^ VILO^™^ Master Mix (#11755050, Invitrogen) in accordance with the manufacturer’s instructions. Then, cDNA was amplified by using the 2x QuantiNova SYBR PCR Master Mix (#208154, Qiagen, Germany) in the QuantStudio 5 Real-Time PCR System (Applied Biosystems, USA) with the following parameters: hold stages at 95°C for 2 min; PCR stages at 95°C for 5 s and 60°C for 10 s; and melt curve stages at 95°C for 15 s, 60°C for 1 min, and 95°C for 1 min. Primers used in this study are β-actin; 5’- TTCTTGGGTATGGAATCCTGT -3’ (forward) and 5’- AGCACTGTGTTGGCATAGAG -3’ (reverse), Runx2; 5’- AGCCTCTTCAGCGCAGTGAC -3’ (forward) and 5’- CTGGTGCTCGGATCCCAA -3’ (reverse), ALP; 5’- TCCTGGCTCTGCCTTTATTCC -3’ (forward) and 5’- TGCCCAAGAGAGAAACCTGCT -3’ (reverse), OPN; 5’- CCCATCTCAGAAGCAGAATCTT -3’ (forward) and 5’- GTCATGGCTTTCATTGGAGTTG -3’ (reverse), OCN; 5’- AAGCAGGAGGGCAATAAGGT (forward) and 5’- TTTGTAGGCGGTCTTCAAGC -3’ (reverse). Fold change was calculated based on mRNA expression using the normalized 2^−ΔΔCt^ method.

### 2.12 Western blot analysis

Western blot analysis and immunofluorescence were used to check the involvement of the p-ERK1/2 signaling pathway in the presence of each material. Previously it was reported that p-ERK1/2 is activated at early time points of material treatments [16]. Because of this, we treated the cells with each material for 2 h. After treatment, cells were washed and lysed with RIPA buffer (Biosesang, South Korea) mixed with protease inhibitor (#04693124001, Roche, Switzerland) and phosphatase inhibitor (#P3200-001, GenDEPOT, USA). The proteins were separated by 12% sodium dodecyl sulfate-polyacrylamide gel electrophoresis, transferred to a polyvinylidene fluoride (PVDF) membrane, and blocked with 5% BSA at 4°C overnight. Next, the membrane was treated with primary antibodies (phospho-p44/42 MAPK [#9101, Cell Signaling, USA, 1:1000], p44/42 MAPK [#9102, Cell Signaling, 1:1000], β-Actin rabbit mAb [#4970S, Cell Signaling, 1:5000]) and then with secondary antibody for 1 h at RT. The bands on PVDF were detected with a LAS 4000 device (Fujifilm Life Sciences, USA) using the Clarity Western ECL Substrate (Bio-Rad, USA). Finally, the p-ERK1/2/ERK1/2 ratio was quantified by ImageJ version 1.53k.

### 2.13 Immunofluorescence

After treatment with each material for 2 h, the cells were washed and fixed with 4% paraformaldehyde. The cells were permeabilized with 0.2% Triton X-100 for 15 min at RT and blocked with 3% BSA for 1 h at RT. The cell layer was then incubated with 1) phospho-p44/42 MAPK (#9101, Cell Signaling, 1:200) for 1 h at RT, 2) secondary antibody (#ab150077, Abcam, USA, 1:500) for 1 h at RT, and 3) 1× phalloidin (#A20009, Invitrogen) for 1 h at RT. Finally, the cell layer was observed under a fluorescence microscope.

### 2.14 Enzyme-linked immunosorbent assay

To confirm the involvement of the ERK1/2 signaling pathway, we performed blockage of the signaling by using a selective inhibitor for the pathway (U0126, Merck) and measured osteogenic protein level. MC3T3-E1 cells were pretreated with U0126 with the concentration of 30 μM for 24 h and induced to differentiate as the previous method in Section 2.6. After 4 days, the OPN protein level was detected with Mouse OPN ELISA Kit PicoKine^®^ (EK0483, Boster Biological Technology, USA) according to the manufacturer’s instruction.

### 2.15 Animals

Twenty male Wistar rats (*Rattus norvegicus*) (250–300 g) provided by the experimental animal center of the Faculty of Pharmacy Airlangga University were used for the animal study. The rats were acclimatized to the laboratory setting and housed under laboratory conditions with a 12-h light/dark cycle. The in vivo study protocol was approved by the Animal Ethics Committee, Faculty of Veterinary Medicine, Airlangga University and complied with the Guide for the Care and Use of Laboratory Animals (National Institutes of Health Publication No. 85–23, revised 1996) and ARRIVE guidelines. The animals were divided into five groups (n = 4): nanoBHA, nanoHA, BHA, HA, and control (no material). The operation procedures were adapted from our previous study [12]. Briefly, ketamine (35 mg kg^−1^, intraperitoneal [i.p.]) and xylazine (2.5 mg kg^−1^, i.p.) were used to anesthetize the animals. The skulls of animals were shaved and applied with an alcohol swab. Next, the skin and periosteum were incised and retracted. Calvarial bone was drilled with a diameter of 4.2 mm, and each material was applied to the defect site. The wound was sutured, cleaned with povidone-iodine, and wrapped with gauze. To prevent infection, ampicillin (25 mg kg^−1^, i.p.) was administered, and wound care was provided for 7 days. The rats were sacrificed via cervical dislocation 6 weeks after the day of operation. Calvarial bone was then stored in a 10% formalin solution for further analysis.

### 2.16 Micro-Computed Tomography (Micro-CT)

The bone samples were scanned using a micro-CT scanner (Skyscan 1176, Bruker) with a resolution of 25 μm and visualized using CTVox (Bruker).

### 2.17 Histology

Calvarial bone was decalcified using 10% formic acid (#100264, Merck, USA), formed as a paraffin block, and cut into 4–6-μm sections. The histology section was used to perform hematoxylin-eosin and Masson’s trichome staining in accordance with standard procedures and observed under a microscope (ECLIPSE Ts2R, Nikon, USA).

### 2.18 Statistical analysis

One-way analysis of variance, Kruskal–Wallis test, or Mann–Whitney U test (*α* = 0.05) was used to evaluate statistical significance. All statistical analyses were performed using SPSS version 24.0 (IBM Corporation, Armonk, NY, USA).

## 3. Results

### 3.1 Fabrication and characterization of NanoBHA

**Fig 1:**
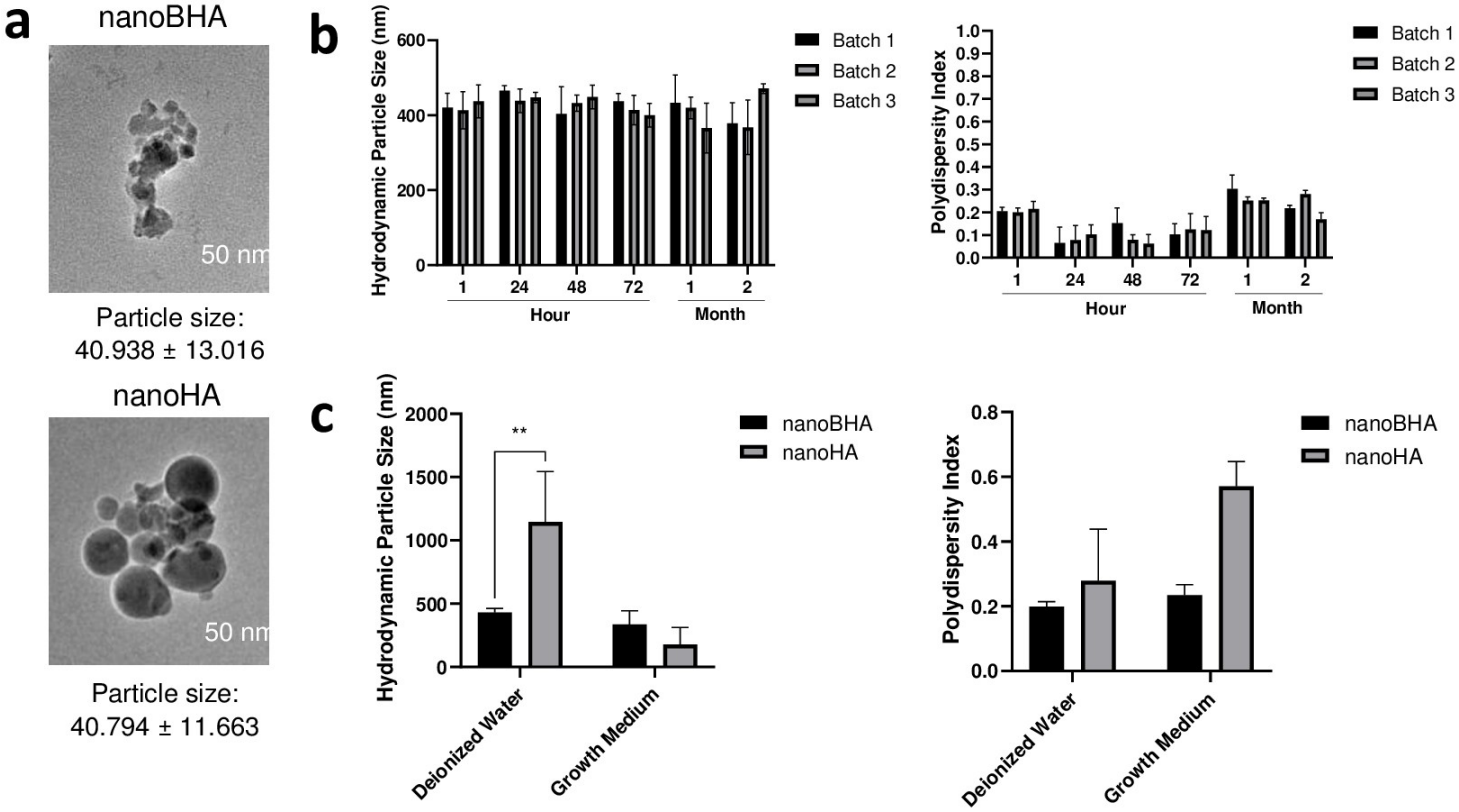
Grain sizes of nanoBHA and nanoHA based on transmission electron microscopy (a). Hydrodynamic particle size and polydispersity index of nanoBHA after fabrication using the wet ball milling method (b). Comparison of nanoBHA and nanoHA on deionized water and growth medium (c).

**Fig 2:**
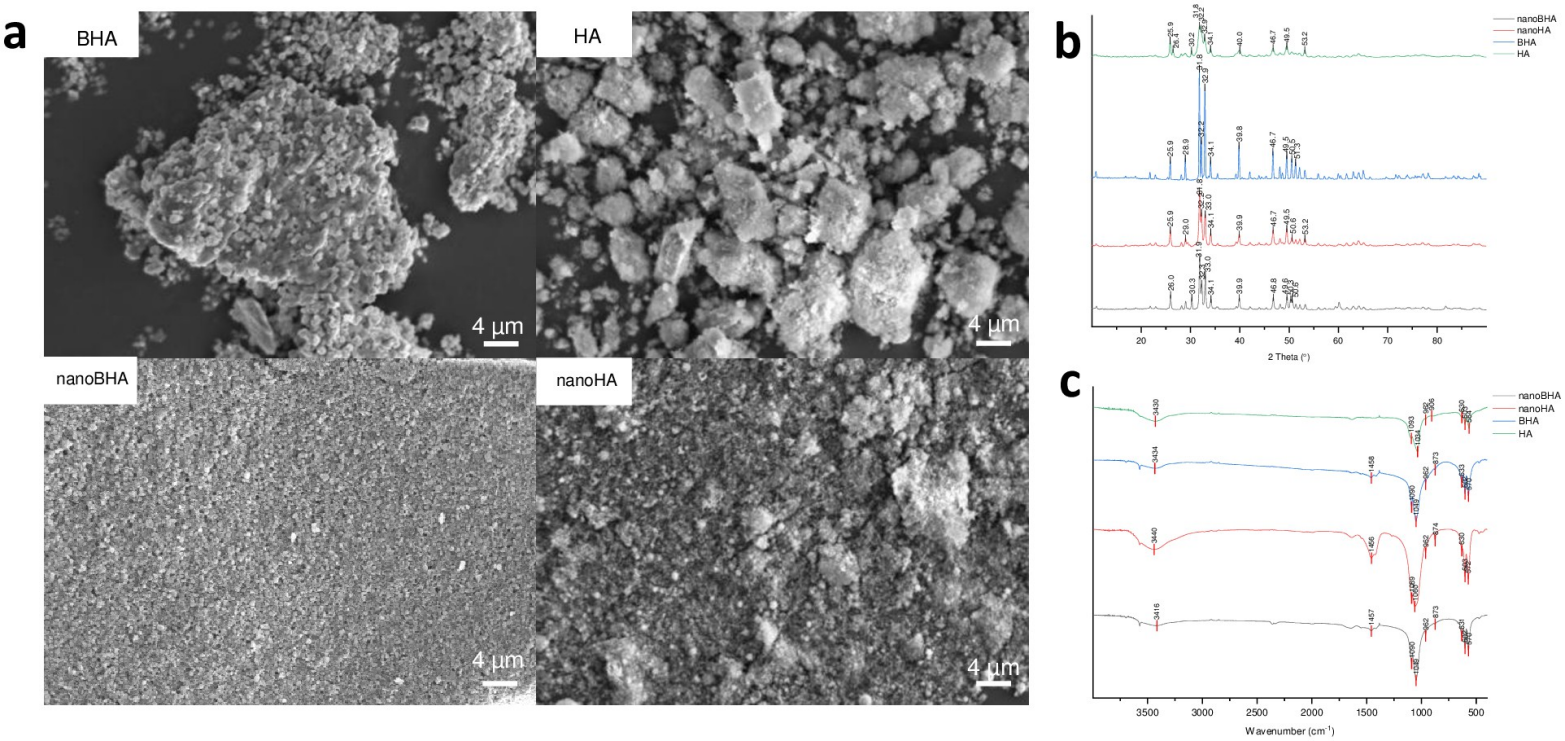
Scanning electron microscopy images (a). XRD (b) and FTIR spectra (c) of BHA, HA, nanoBHA, and nanoHA.

NanoBHA was fabricated using the wet ball milling method. The grain size of nanoBHA was ~40 nm, indicating the successful fabrication of the nanomaterial (Fig 1A). DLS showed that the hydrodynamic particle size of nanoBHA was stable after 2 months of fabrication (Fig 1B), and there was no difference in size from commercially available nanoHA (Fig 1C). The measurement results in growth medium showed that these two nanomaterials had the same size (Fig 1C). This finding indicates that these two nanoparticles will not agglomerate during the in vitro study. Moreover, BHA and HA had micrometer-sized particles with irregular forms (Fig 2A). NanoBHA was shaped irregularly and had similar morphology to BHA, whereas nanoHA had a round shape (Fig 1A). All materials showed the same peak position with different intensities (Fig 2B) and had hydroxyl, carbonate, and phosphate substitution groups, resembling the characteristics of HA (Fig 2C). All materials had similar calcium, phosphorous, and carbon elemental weight percentages, except for nanoBHA and BHA, which had a slightly higher percentage of magnesium and sodium (Table 1). In addition, the zeta potential charge was different in natural and synthetic HA (Table 2).

**Table 1:** Elemental weight of nanoBHA, nanoHA, BHA, and HA (%).

Material	Calcium (Ca)	Phosphorous (P)	Carbon (C)	Magnesium (Mg)	Sodium (Na)
nanoBHA	39.130	18.660	4.250	0.500	0.500
nanoHA	38.454	12.952	4.719	0.100	0.100
BHA	37.638	13.413	7.407	0.400	0.500
HA	25.000	10.800	7.600	0.200	0.100

**Table 2:** Zeta potential value of nanoBHA, nanoHA, BHA, and HA (mV).

Medium	Material			
	nanoBHA	nanoHA	BHA	HA
DI	−13.100 ± 1.210	3.234 ± 0.221	−12.240 ± 0.948	1.674 ± 0.267
Growth medium	−4.680 ± 0.223	−8.102 ± 0.668	−7.860 ± 0.613	−8.824 ± 0.481

### 3.2 Viability and proliferation capacity of NanoBHA compared with other HAs

**Fig 3:**
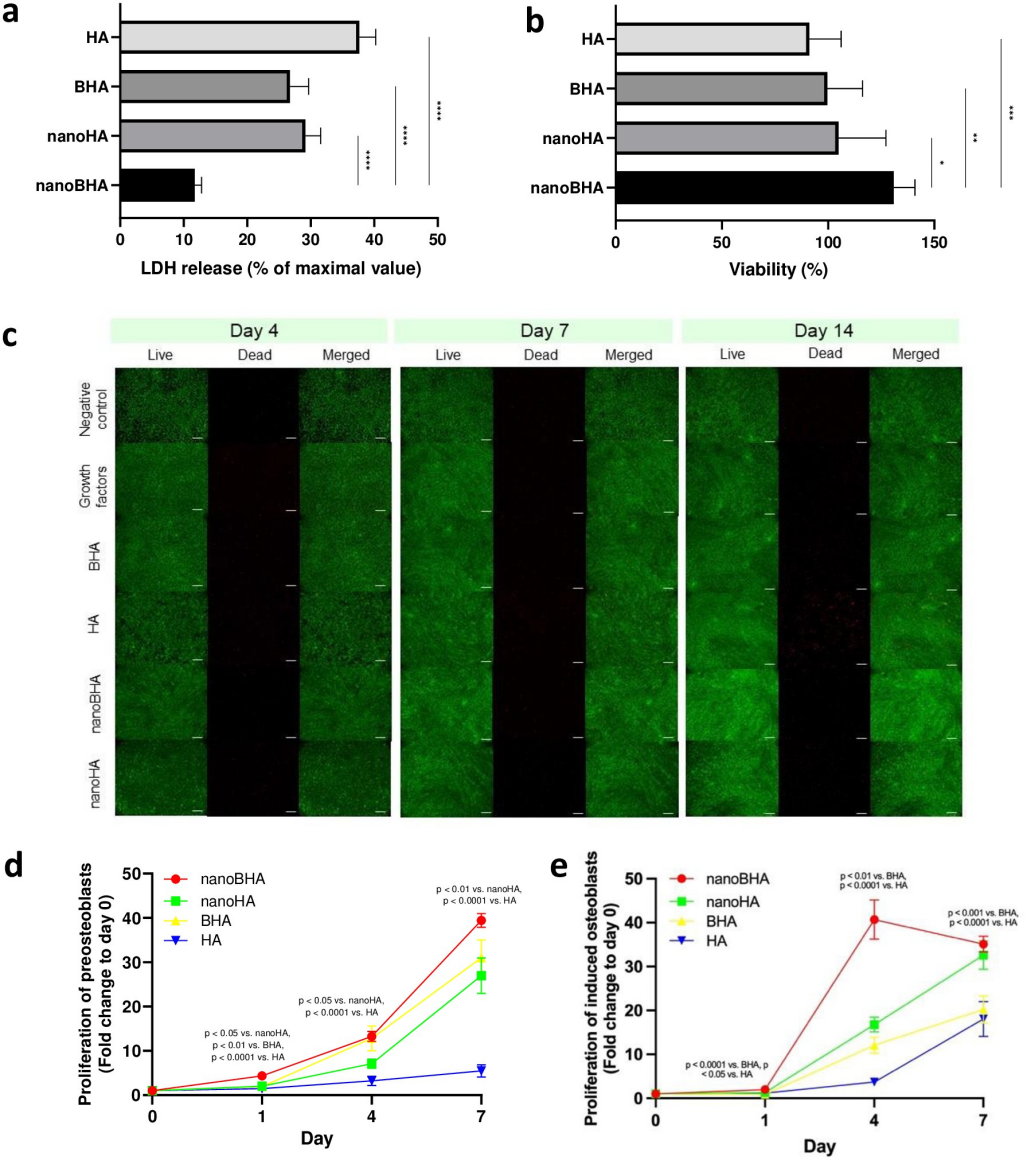
LDH release (a), cell viability (b), live/dead cell viability (c), and proliferation of preosteoblasts (d) and induced osteoblasts (e) from nanoBHA, nanoHA, BHA, and HA groups. Scale bar: 200 μm.

The viability of all materials was examined using LDH, CCK-8, and live/dead assays. Compared with other groups, the nanoBHA group had the lowest percentage of LDH release, with an LDH percentage of 11.723 ± 1.089 (Fig 3A). The viability of nanoBHA was 130.853 ± 10.061% and significantly higher than that of other groups (Fig 3B). Moreover, the viability of all materials was examined in differentiation induction conditions to ensure that these conditions would have no toxic effect. Live/dead assay showed that the number of live cells was remarkably higher than that of dead cells in all groups and at all time points, and no difference in live/dead cells was observed in the negative control and growth factor groups (Fig 3C). This finding proved that the developed condition did not induce cell death. However, on day 14, the number of dead cells was remarkably higher in the HA group than in other groups (Fig 3C), indicating that HA induced cell death at later time points. Furthermore, the proliferation assay showed that the nanoBHA group experienced cell proliferation at most time points. This occurred not only in the preosteoblast cell model (Fig 3D) but also in the previously induced cell model (induced osteoblasts, Fig 3E).

### 3.3 Osteogenic differentiation capacity of NanoBHA compared with other HAs based on osteoblast phenotype and calcium deposition

**Fig 4:**
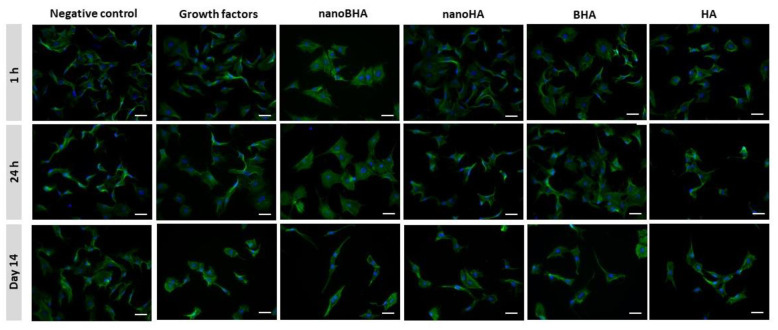
Morphology of preosteoblasts after 14 days of differentiation induction. Green, F-actin; blue, nuclei. Material concentration = 50 μg/mL. Scale bar: 50 μm.

**Fig 5:**
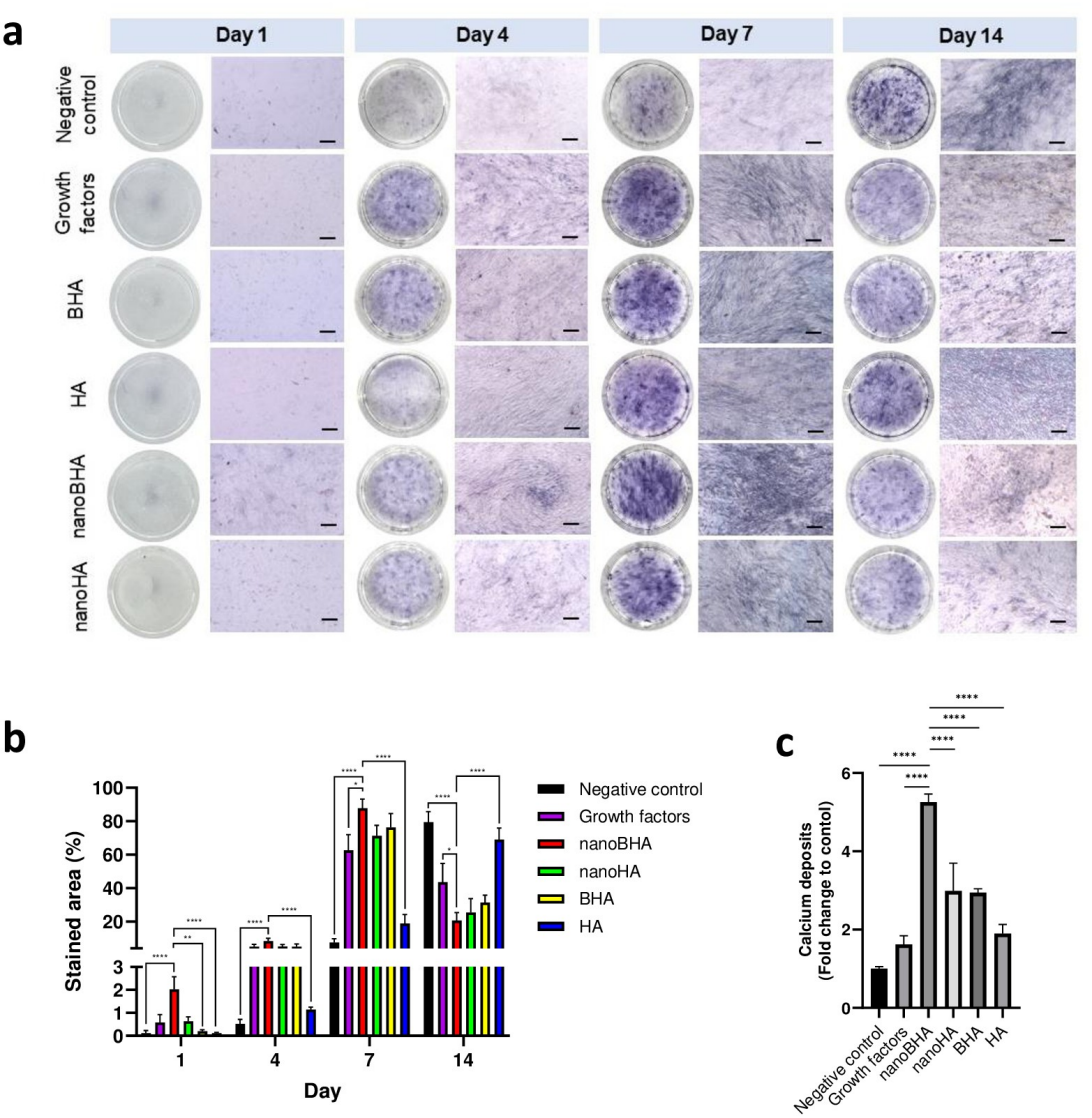
ALP staining results (a) and the corresponding quantification (b) and calcium deposition of osteoblasts after 14 days of differentiation inducement (c). Scale bar: 10 μm.

The ability of all materials to induce osteogenic differentiation was assessed using staining assays to observe cell morphology and ALP enzyme and calcium deposits. At early time points, all groups had the same morphology as preosteoblasts, characterized by unorganized F-actin, except for the nanoBHA group. On day 14, the negative control group showed no morphological changes, whereas other groups, especially the nanoBHA group, experienced morphological changes with organized F-actin resembling mature osteoblasts (Fig 4). Moreover, ALP staining showed robust ALP detected on day 7 (Fig 5A), with the highest stained area in the nanoBHA group (Fig 5B). On day 14, the percentage of the stained area in the nanoBHA group decreased, whereas the percentage in the negative control and HA groups peaked (Fig 5A and 5B). ALP is an enzyme that marks the early stage of osteogenic differentiation. Typically, ALP activity will peak in the early stages of differentiation and decrease in the final stages. In this study, nanoBHA accelerated osteogenic differentiation based on the phenotype of mature osteoblasts. Furthermore, the ability of cells to deposit calcium was examined 14 days after the induction of differentiation by each material. The results showed that the nanoBHA group had a higher fold change of calcium deposits compared with other groups (Fig 5C). This finding proved that nanoBHA was the best material for inducing osteoblasts to deposit calcium.

### 3.4 Osteogenic differentiation capacity of NanoBHA compared with other HAs based on the expression of osteogenic genes

**Fig 6:**
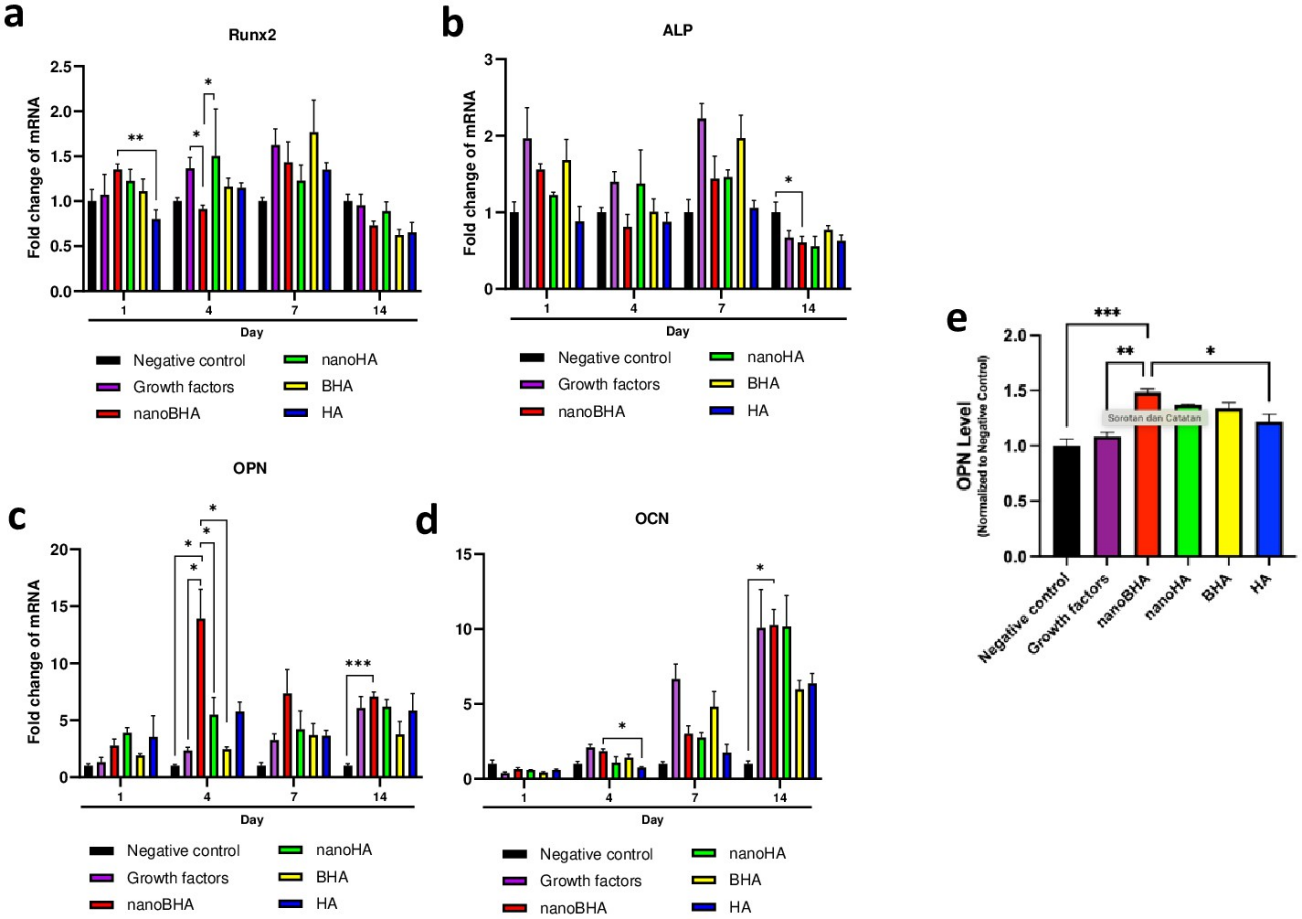
Gene expression of osteogenic differentiation markers: Runx2 (a), ALP (b), OPN (c), and OCN (d), as well as OPN protein level (e).

The expression of Runx2, ALP, osteopontin (OPN) and osteocalcin (OCN) genes was assessed to examine the stages of osteogenic differentiation. Runx2 and ALP are typically expressed in the early stages of differentiation and reduced when the cells enter the final stages, whereas OPN and OCN show the opposite trend. The expression of Runx2 was higher in the nanoBHA group than in the HA group on day 1. However, on day 4, the expression of Runx2 was lower in the nanoBHA group than in other groups (Fig 6A). By contrast, the expression of ALP was lower in the nanoBHA group than in the negative control group on day 14 (Fig 6B). Differences in expression between groups became increasingly visible for non-collagen protein genes, including OPN and OCN. On day 4, the expression of OPN was higher in the nanoBHA group than in other groups. A similar finding was also observed on day 14 (Fig 6C). Moreover, on day 14, the expression of OCN was higher in the nanoBHA group than in the negative control group (Fig 6D). Furthermore, we validated the gene expression level. Since OPN is the gene that underwent the most significant change by qRT-PCR, we measure OPN protein level with the ELISA technique. Results proved that the OPN protein level was in line with its gene expression (Fig 6E). These results showed that nanoBHA affected the acceleration of osteogenic differentiation by rapidly expressing and decreasing the expression of early differentiation markers and increasing the expression of late differentiation markers.

### 3.5 Effects of NanoBHA on the ERK1/2 signaling pathway compared with other HAs

**Fig 7:**
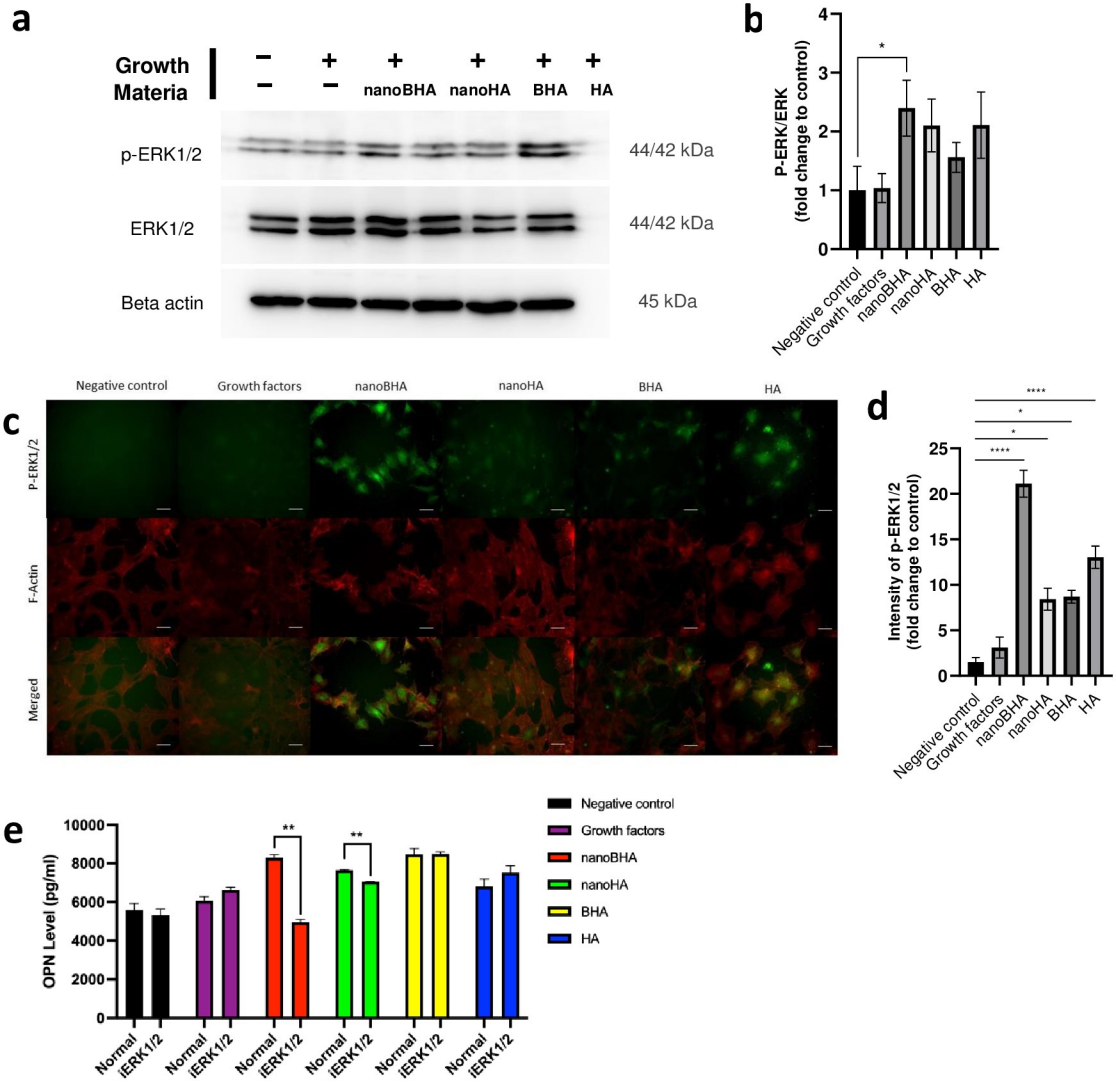
Results of Western blot analysis of p-ERK1/2 and ERK1/2 (a) and the corresponding quantification of p-ERK/ERK (b). Immunofluorescence imaging of p-ERK1/2 (c) and the corresponding quantification of p-ERK1/2 intensity (d). OPN protein level, with and without pretreatment of ERK1/2 signaling pathway inhibitor (e). The quantifications were based on fold change from the negative control. Scale bar: 50 μm. Blots from b were observed for each antibody as separated in white space, the original blots are presented in S1 Raw image.

Western blot analysis and immunofluorescence were conducted to examine the involvement of the ERK1/2 signaling pathway when cells were treated with each material. The results of Western blot analysis showed that the nanoBHA group had a higher p-ERK/ERK ratio compared with the negative control group (Fig 7A and 7B). This finding is supported by the immunofluorescence results, which showed a high fluorescence intensity in the nanoBHA group (Fig 7C and 7D). Moreover, we proved the involvement of ERK1/2 signaling pathway by blocking this pathway using a selective inhibitor and measured the OPN protein level. Results demonstrated that this signaling was involved in the OPN synthesis induced by nanoBHA (Fig 7E). This finding indicates that the administration of nanoBHA activates the ERK1/2 signaling pathway in preosteoblasts, which causes cellular changes in the cells, including OPN synthesis.

### 3.6 In vivo performance of NanoBHA compared with other HAs in rat calvarial defect

**Fig 8:**
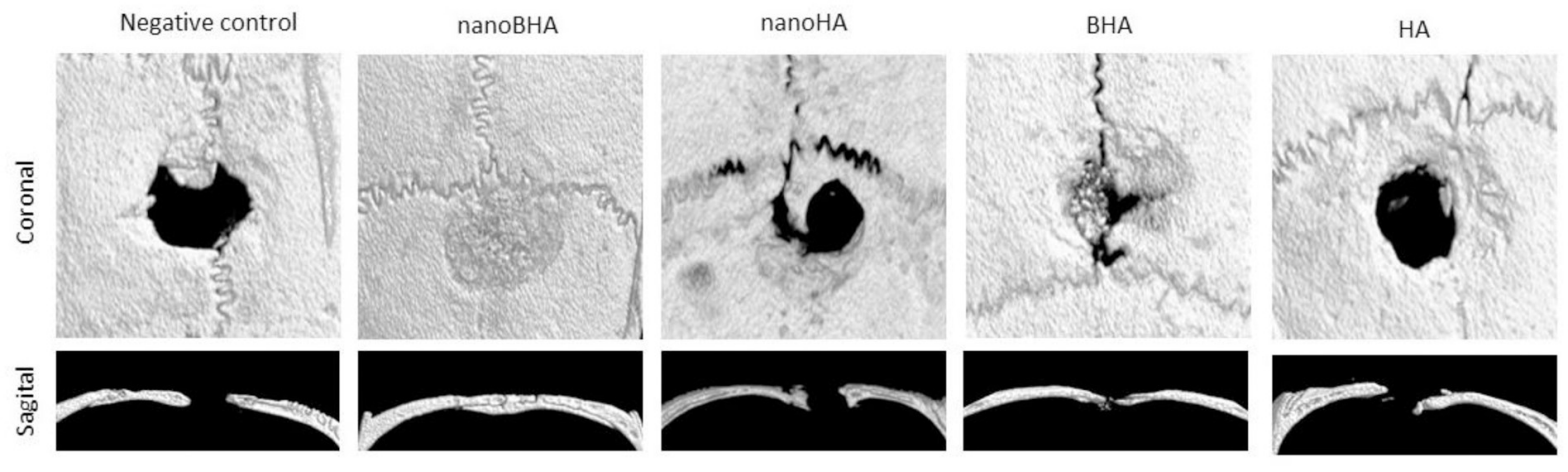
Representative micro-CT images of the augmented region after 6 weeks of treatment. Scale bar: 100 μm.

**Fig 9:**
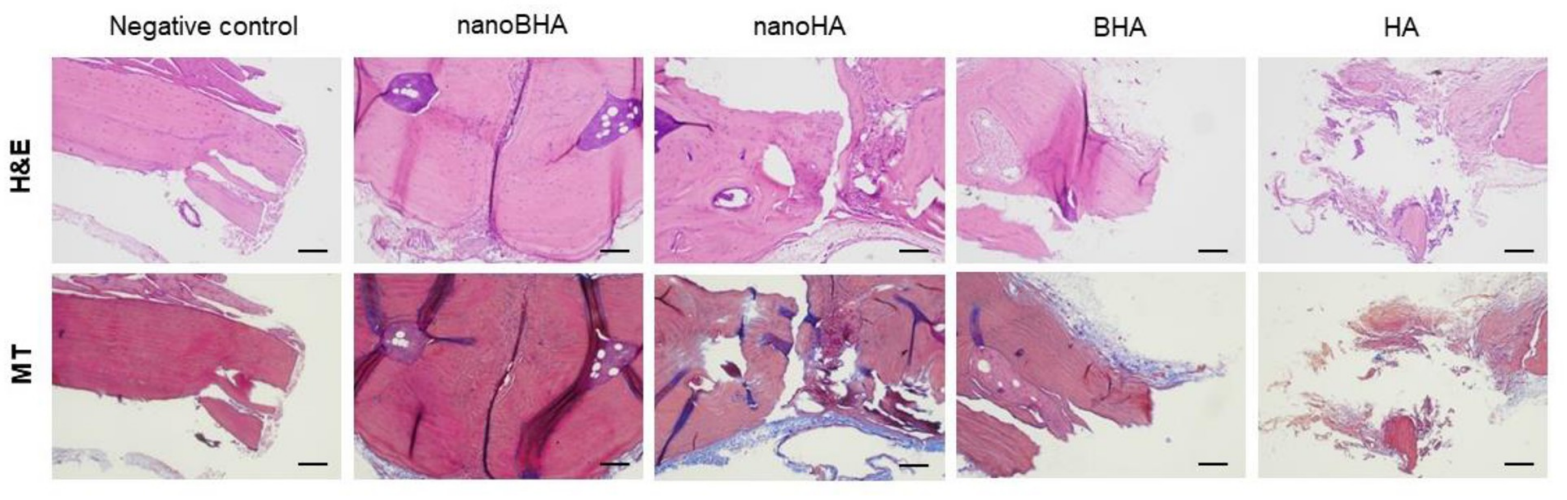
Representative histological images of the augmented region after 6 weeks of treatment. H&E, hematoxylin–eosin; MT, Masson’s trichrome. Scale bar: 100 μm.

The performance of nanoBHA was evaluated in vivo using the rat calvarial defect to mimic the condition of a critical-sized bone defect. Six weeks after the day of implantation, the calvarial bone was taken and washed properly before evaluation to eliminate the remaining materials. The CT images showed that groups treated with nanoBHA qualitatively showed bone growth in the defect site (Fig 8). This finding was also supported by the histological results. Bone cells and connective tissue were found at the nanoBHA implantation site (Fig 9). These results confirm that nanoBHA has superior in vivo action, which correlates with the in vitro results.

## 4. Discussion

This study investigated the effects of nanosized BHA (nanoBHA) on preosteoblast maturation and in vivo bone regeneration compared with other HAs. NanoBHA was fabricated by mechanically reducing the particle size of BHA. In a previous study, we used the dry ball milling method to reduce the particle size of BHA. After 9 h of milling, the particle size was reduced from ~5 μm to ~1 μm. However, the method was ineffective for producing nanomaterials because no media were used in the milling process [18]. In a previous study, ethanol was used as a milling medium to reduce the particle size of BHA [21]. Ethanol has been used as a PCA in the milling process [20]. Nevertheless, ethanol and other PCAs may affect the fabricated material, including changes in particle morphology and elemental distribution [19, 20]. In the present study, deionized water was used as a milling medium to fabricate nanoBHA. The results showed that the hydrodynamic particle size of nanoBHA was reduced to ~400 nm with a grain size of ~40 nm.

The milling treatment used in the present study did not change the material’s morphology. A previous study reported that the regular particle shape of calcium phosphate gave better cell viability, osteogenic gene expression, drug loading, and antibacterial effect compared with flaky, brick-like, and elongated orthogonal particles [22]. However, the regular-shaped material had the smallest particle size, which was around 20 nm compared with that of other materials that were on the micrometer scale [22]. Thus, this finding indicates that the activity produced was also influenced by particle size.

Aside from having the same morphological appearance, nanoBHA and BHA also had hydroxyl, carbonate, and phosphate groups that were also detected in other HAs, animal-based HA [23], and human bone [24]. On the basis of elemental weight, calcium contributed the highest weight percentage to all materials. Meanwhile, on the basis of electrokinetic potential, all materials had negative charges on the growth medium, which were predicted to be influenced by the proteins contained in the growth medium [16, 25]. Aside from particle size, crystallinity was another feature that distinguished nanoBHA and HA. The particle size is closely related to the material’s crystallinity: a decrease in particle size causes a broadening of the XRD peak [25]. Thus, because of the particle size of nanoBHA, it had a lower peak intensity compared with BHA. This study proved that the wet ball milling method was able to reduce the particle size of BHA to the nanoscale without changing its characteristics.

In addition, we determined the effects of nanoBHA on preosteoblasts. All HAs used in the study had a cell viability >70%, indicating their nontoxicity [26–28]. However, the type and size of HAs had different effects on LDH release, with nanoBHA having the lowest LDH release. Moreover, at later time points in differentiation conditions, synthetic HA induced greater cell death. This finding may be because of the impurity resulting from the chemical synthesis of HA. HA impurities, including cadmium, can cause the accumulation of Exportin-1 and phosphorylation of Jnk, leading to DNA damage and cell apoptosis [29]. Moreover, our study proved that nanoBHA was the most effective material in inducing cell proliferation in preosteoblasts and osteoblasts. Nanomaterials are widely known to induce better cell proliferation compared with micromaterials [30–32]. This effect is not only related to the particle size but also to the material’s nano-/microsurface topography [32].

Furthermore, nanoBHA exhibited better cell differentiation and calcium deposition activities compared with nanoHA, BHA, and HA. The difference in particle size was the main reason for nanoBHA’s superiority. Several theories have been developed regarding the effect of the material’s particle size on osteoblast activity. The first is the possibility of nanoparticle uptake by osteoblasts [33–35]. Studies have found that nanoparticles are endocytosed by osteoblasts through the change of LC3β-I to LC3β-II, resulting in cell differentiation and calcium deposits [35, 36]. The second is the role of specific receptors on osteoblasts. A previous study observed an increase in osteogenic differentiation markers when preosteoblasts were treated with synthetic HA nanoparticles [16]. Moreover, when the membrane proteins Fgfr and PiT were blocked, differentiation markers failed to increase. This finding demonstrates that nanoparticles may have specific interactions with osteoblast receptors [16]. In the present study, OPN is a regulator that plays an important role in the molecular processes that occur because of nanoBHA. OPN was previously reported to act as a specific regulator of osteoblasts in the presence of phosphorus [37] and calcium [38]. In another study, OPN was also found to be a specific regulator when preosteoblasts were treated with synthetic HA nanoparticles [39]. On the basis of these findings, the particle size of HA tends to have a specific influence on osteoblasts’ molecular regulation by OPN.

Another interesting finding in the present study was that although nanoBHA and nanoHA were present at the nanoscale, nanoBHA had higher levels of proliferation, differentiation, and calcium deposition compared with nanoHA. This finding demonstrates that natural HA provides better osteoblast activity compared with synthetic HA. Natural HA is a nonstoichiometric HA that contains various trace elements including Na^+^, Sn^2+^, Mg^2+^, K^+^, Ba^2+^, F^−^, and CO_3_^2−^, providing it with chemical compositions similar to those of human bones [40, 41]. Moreover, the source and method of extraction provide different elements with varying weight percentages compared with natural HA, which is difficult to imitate in synthetic HA [42]. Here, we found that natural HA had Mg and Na elements compared with synthetic HA. This finding is consistent with a previous report [10]. Mg and Na are known to exert superior activity on various osteoblast-derived cells, including induction of cell proliferation and differentiation [43–45].

In addition, we also explored the involvement of the ERK1/2 signaling pathway in the presence of treated materials. ERK1/2 signaling has been reported to mediate the cellular events of osteoblasts provided by HA-based materials [46], including micro-/nanohybrid surface HA [47] and HA nanoparticles [16, 48]. Because of this, we hypothesized that this signal also plays a role in nanoBHA treatment. Our hypothesis was proven by the findings of the study that showed that ERK1/2 was activated in preosteoblasts when the cells were treated with nanoBHA. ERK1/2 is a protein that plays a role in several molecular events in osteoblasts, including proliferation and differentiation [49]. The results of the present study indicated that the proliferation and differentiation of osteoblasts caused by nanoBHA may be mediated by the ERK1/2 signaling pathway.

Furthermore, our in vivo study demonstrated that nanoBHA induced bone growth in the defect area of experimental animals, consistent with the in vitro findings. In addition to the aforementioned factors, this is also influenced by physiological conditions when materials come in contact with bone tissue. Protein adsorption is one way for bone tissue to recognize biomaterials. Compared with microsized materials, the smaller size of nanoBHA makes it have a larger surface area. This provides the material with high wettability, which is usually indicated by a lower contact angle [13, 50]. The high wettability of the material will help osteoblasts adhere to HA through RGD peptides present in osteoblasts and speed up the bone regeneration process [13, 51].

This study has several limitations. For example, the involvement of membrane proteins in the molecular mechanism of nanoBHA on osteoblasts was not investigated. However, this study has revealed the mechanism of preosteoblasts when cells are exposed to different types and sizes of HA. We expect that these findings will lead to more studies on nanomaterials and their mechanisms of action in bone remodeling.

## 5. Conclusions

Here we reported that the wet ball milling method was able to reduce the size of BHA particles to the nanometer scale without changing its natural characteristics, including morphology, chemical substitution group, and elemental composition. Compared with nanoHA, BHA, and HA, nanoBHA provided superior performance. (i) NanoBHA could maintain the viability of preosteoblasts based on LDH release, dehydrogenase enzyme activity, and reactions with live/dead reagent. (ii) NanoBHA could induce the proliferation of preosteoblasts and osteoblasts. (iii) NanoBHA accelerated the differentiation of preosteoblasts to osteoblasts based on cell morphology, ALP expression, and osteogenic genes expressions. (iv) NanoBHA could induce the calcium deposition of cells. (v) At the molecular level, treatment with nanoBHA and HA activated the ERK1/2 signaling pathway, which is associated with the proliferation/differentiation and cell death of osteoblasts, respectively. (vi) NanoBHA induced bone growth in the defect area of experimental animals, which was consistent with the in vitro findings.

This study also revealed that OPN was one of the regulators that play an important role in the molecular processes that occur because of nanoBHA. Moreover, despite being present in the nanoscale, natural HA provided better cell proliferation, differentiation, and calcium deposition compared with synthetic HA, which may be because of the materials’ intrinsic chemical properties.

This study discovered the mechanism of preosteoblasts exposed to different types and sizes of HA. These findings may generate interest with regard to studies on nanomaterials and their mechanisms of action in bone remodeling.

## Supporting information

S1 Raw image (PDF)

S1 File (DOCX)
