# Supplementary material for: Comparative study of bovine and synthetic hydroxyapatite in micro- and nanosized on osteoblasts action and bone growth
Source: PLoS One. 2025 Jan 24;20(1):e0311652. doi: 10.1371/journal.pone.0311652 (PMC11759404; doi:10.1371/journal.pone.0311652)

Original blots from Figure 7a.

1. Raw original blot stained with p-ERK1/2 antibody.
   Loading order (same in all images, from left to right), negative control, growth factors, nanoBHA, nanoHA, BHA, HA.
   The method used to capture images: The bands on PVDF were detected with a LAS 4000 device (Fujifilm Life Sciences, USA) using the Clarity Western ECL Substrate (Bio-Rad, USA). Finally, the p-ERK1/2/ERK1/2 ratio was quantified by ImageJ version 1.53k.

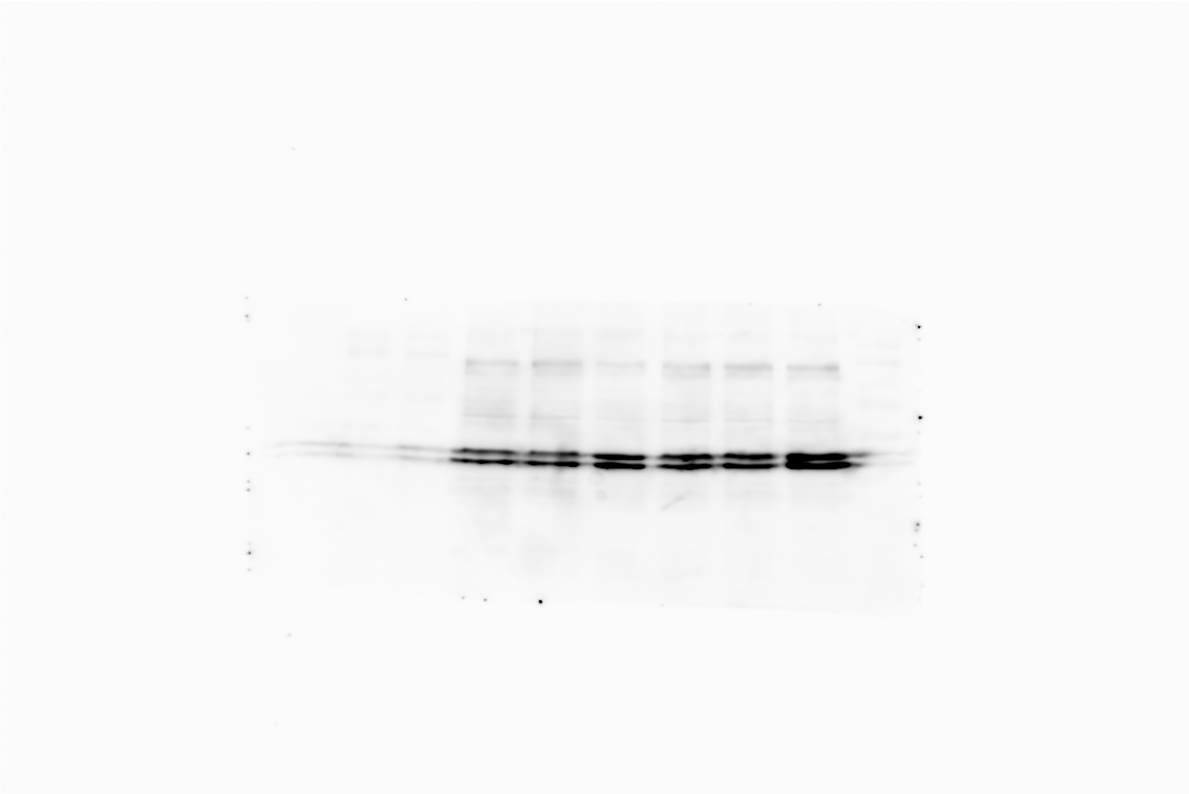

2. Raw original blot stained with ERK1/2 antibody.
   Loading order (same in all images, from left to right), negative control, growth factors, nanoBHA, nanoHA, BHA, HA.
   The method used to capture images: The bands on PVDF were detected with a LAS 4000 device (Fujifilm Life Sciences, USA) using the Clarity Western ECL Substrate (Bio-Rad, USA). Finally, the p-ERK1/2/ERK1/2 ratio was quantified by ImageJ version 1.53k.

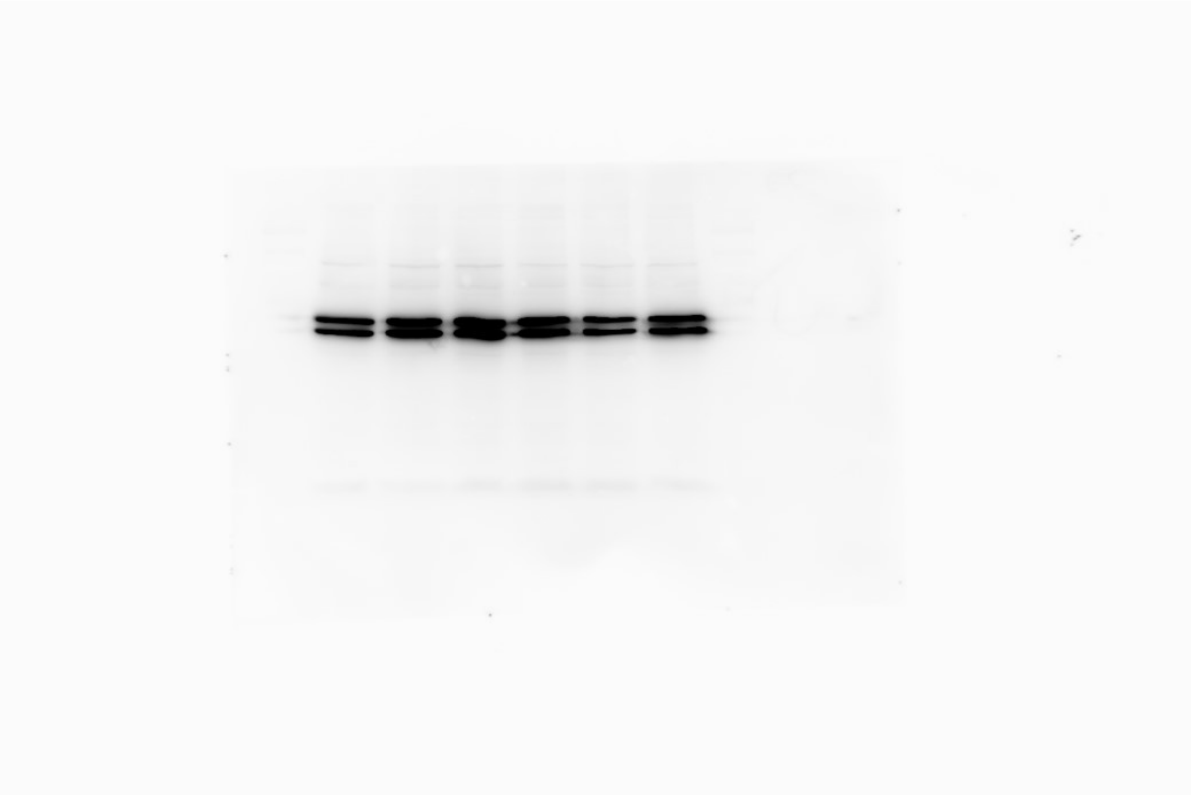

3. Original blot stained with beta actin antibody original and with multiple exposures. Loading order (same in all images, from left to right), negative control, growth factors, nanoBHA, nanoHA, BHA, HA.
   The method used to capture images: The bands on PVDF were detected with a LAS 4000 device (Fujifilm Life Sciences, USA) using the Clarity Western ECL Substrate (Bio-Rad, USA). Finally, the p-ERK1/2/ERK1/2 ratio was quantified by ImageJ version 1.53k.

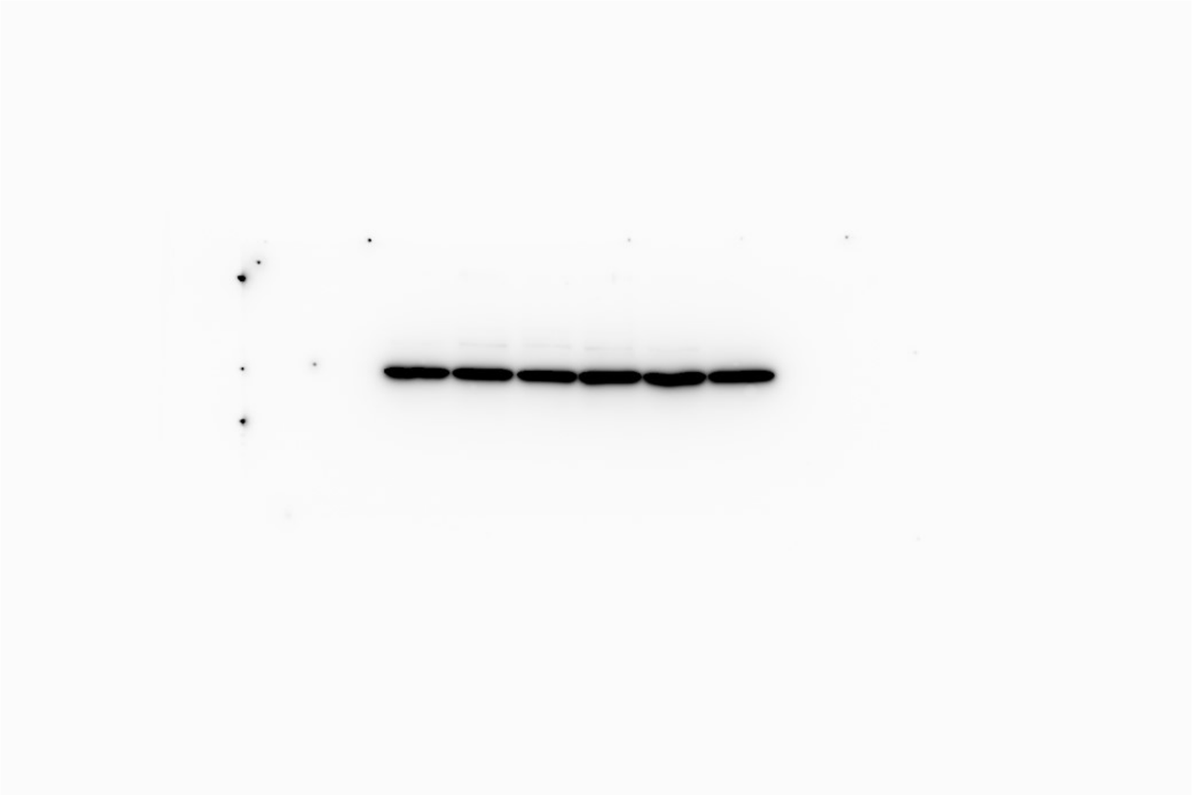

Supplement: S1 Raw image — (PDF) [file pone.0311652.s001.pdf]
