# Supplementary figures and images for: Comparative study of bovine and synthetic hydroxyapatite in micro- and nanosized on osteoblasts action and bone growth
Source: PLoS One. 2025 Jan 24;20(1):e0311652. doi: 10.1371/journal.pone.0311652 (PMC11759404; doi:10.1371/journal.pone.0311652)

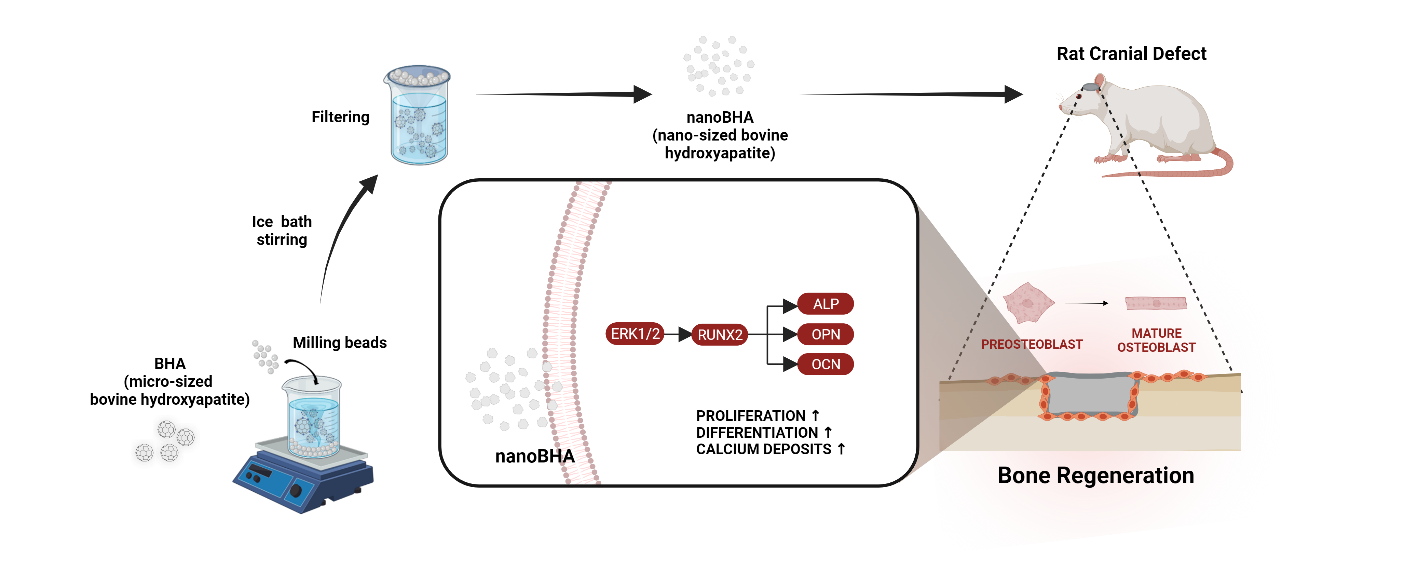

Supplement: S1 File — (DOCX) [file pone.0311652.s002.docx]
